# Prevalence and Determinants of Antibiotic Self-Medication among Adult Patients with Respiratory Tract Infections in the Mboppi Baptist Hospital, Douala, Cameroon: A Cross-Sectional Study

**DOI:** 10.3390/diseases6020049

**Published:** 2018-06-08

**Authors:** Roland Cheofor Ngu, Vitalis Fambombi Feteh, Belmond Tse Kika, Emade Ketchemen Nerice F., Chia Mark Ayeah, Theresia Chifor, Tsi Njim, Alvine Manuela Fankem, Franklin Kwenti Fai Yengo

**Affiliations:** 1 Medical Doctors Research Group (MDRG), Douala 15161, Cameroon; crolandn@cbchealthservices.org (R.C.N.); vitalfeteh@gmail.com (V.F.F.); belmondkika@gmail.com (B.T.K.); emadenericek@gmail.com (E.K.N.F.); ayeahmarkchiatoh@yahoo.com (C.M.A.); 2 Mboppi Baptist Hospital Douala, Douala 15161, Cameroon; chifor.terry@gmail.com (T.C.); manuelafankem@gmail.com (A.M.F.); faiyo.fy@gmail.com (F.K.F.Y.); 3 Health and Human Development (2HD) Research Network, Douala 4856, Cameroon; 4 Nuffield Department of Medicine, University of Oxford, Oxford OX1 3SY, UK; 5 District Hospital Ekondo-Titi, Ekondo-Titi 281, Cameroon; 6 School of Public Health – University of Brussels, Brussels CP 598, B-1070, Belgium; 7 Department of International Public Health, Liverpool School of Tropical Medicine, Liverpool L3 5QA, UK

**Keywords:** self-medication, prevalence, risk factors, antibiotics, respiratory tract infection, Cameroon, Africa, antimicrobial resistance

## Abstract

Antibiotic self-medication in patients with respiratory tract infections (RTI) is increasing globally and has been reported to be one of the prime contributors to antimicrobial resistance (AMR). Our study aims to provide data on the prevalence of antibiotic self-medication and identify the factors contributing to self-medication in adult patients with respiratory tract infection in an urban setting in Cameroon. This was cross-sectional study carried out at Mboppi Baptist Hospital, Douala, Cameroon. A validated structured questionnaire was administered to 308 consenting participants with diagnosed RTIs, to collect data on socio-demographic characteristics and history of antibiotic self-medication. Significance was set at a *p*-value < 0.05. The prevalence of antibiotic self-medication amongst individuals with RTIs was 41.9% (95% CI 36.5% to 47.5%). Patients with a history of pulmonary tuberculosis (TB) were significantly less likely to self-medicate with antibiotics (*p*-value = 0.043). The most common source of antibiotic self-medication was pharmacies (62%) and Cotrimoxazole and Amoxicillin were the most commonly used antibiotics (38.8% (50), 26.4% (34), respectively). Self-medication with antibiotics in adult patients with RTIs is common in Cameroon. Control of the use of antibiotics, organisation of medication stewardship programs, and education of the general population on the adverse consequences of antibiotic self-medication are required.

## 1. Introduction

Antimicrobial drug resistance is rapidly increasing globally and self-medication using insufficient dosages or incorrect or unnecessary drugs is a recognized risk factor of selective antimicrobial resistance [1,2].

Self-medication can be defined as the use of drugs to treat self-recognized disorders or symptoms or the intermittent or continued use of a prescribed drug for chronic or recurrent diseases or symptoms without the advice of a physician [3]. Use without medical guidance is inappropriate because using insufficient dosages or incorrect or unnecessary drugs increases the risk of the selection of resistant bacteria and the spread of antimicrobial drug resistance [4].

Self-medication is commonly practiced worldwide, particularly in developing countries, and is considered as an alternative for people who cannot afford the cost of healthcare services [5,6,7]. Due to lack of access to health care facilities, many individuals opt to receive initial treatment for febrile illnesses at home using herbal medicines, oral antipyretics, and antimalarial or antibiotic drugs purchased in local shops without prescription, which are often substandard or falsified medications, especially in the case of low- and middle-income countries where antimicrobial stewardship is poor [8].

Self-medication can be a major contributing factor towards the inappropriate use of antibiotics in patients with respiratory tract infections (RTI) and, hence, antimicrobial resistance. In addition, recent antibiotic use has been reported as a risk factor for infection or colonization with resistant bacterial pathogens [9]. The increase in resistance to antimicrobial drugs represents an important clinical and social problem [10].

Ivanovska et al. (2013) reported a 17.8% prevalence of antibiotic self-medication in adult patients with respiratory tract infections in the Republic of Macedonia [11]. A study carried out in the United States of America (USA) showed that the most common reasons for self-medication were a common cold and upper respiratory tract symptoms [12], while another carried out in Europe revealed “a sore throat” and bronchitis [10] to be the main inciting factors. The main self-medication sources are pharmacies and medications leftover from previous prescriptions [4].

Risks associated with self-medication include lack of clinical evaluation of the condition by a health care provider which could result in misdiagnosis and incorrect choice of drugs, delays in seeking appropriate treatment, use of excessive or substandard drugs and prolonged durationof use, drug interactions, and polypharmacy [13,14]. All these play a pervasive role in the development of antibiotic-resistant microbes. The determinants of self-medication with antibiotics in low- and middle-income countries include unregulated importation and sale of over-the-counter antibiotics, the cost of medical consultation, low satisfaction with medical practitioners, and misconceptions regarding the efficacy of antibiotics [15,16,17]. Another risk factor for actual self-medication is the availability of drugs at home, which encourages use [4], while cultural beliefs and a lack of health insurance are other possible determinants of self-medication [12].

In Cameroon, drug use and misuse has been described by Sjaak Van der Geest, who reported on pharmaceutical use in the South West Region in the 1980s. He attributed the inappropriate use of drugs in Cameroon to be due to the ineptitude of the state-run healthcare facilities, which were at that time considered by Cameroonians to be under-staffed due to “frequent absences of health workers”, “characterized by bureaucratization and poor management” and recurrent medication shortage [18]. By 1995, a World Bank report noted that instead of going immediately to the formal sector when ill as was the practice before 1987, Cameroonians choose to first self-medicate. Other options included visiting a “quack doctor”, street vendor, or a traditional or faith healer, and often, only when very ill would people resort to the hospital (World Bank 1995). Each of these factors has exacerbated inappropriate drug use in Cameroon [18].

To date, the information on self-medication in Africa is limited. In addition, little information exists on factors that put persons at risk for self-medication. Furthermore, antibiotic self-medication practice is under-evaluated among African patients with symptoms and signs of RTI [10]. In Cameroon specifically, few studies have been done with respect to self-medication practices, like in the field of dentistry [19], but there is paucity of data with regards to self-medication in patients with RTI and symptoms of RTI. Thus, our study aims to provide data on the prevalence of antibiotic self-medication in adult patients with RTI and identify the factors influencing this practice in an urban hospital in Cameroon.

## 2. Materials and Methods

### 2.1. Study Design and Setting

This was a cross-sectional hospital-based study carried out in Mboppi Baptist Hospital, Douala (MBHD) in the Littoral region of Cameroon over a period of 6 months (January 2017 to June 2017). It is a faith-based hospital in an urban city of Cameroon. It is made up of an outpatient department, HIV/AIDS treatment centre, operating theatre, and wound management, surgical, obstetric and gynaecological, paediatrics, internal medicine, counseling, pharmacy, laboratory, imaging, and administrative units.

### 2.2. Study Participants and Sampling

The sample population comprised patients aged 21 years and above who presented at MBHD during the study period with symptoms of RTI, whom we recruited consecutively from 1 January to 30 June 2017 after informed consent. Patients with heart failure whose symptoms may mimic respiratory tract infections were excluded from the study.

### 2.3. Sample Size

The sample size was calculated using the Lorenz’s formula n = z^2^ (p) × (q)/d^2^ [20], where p is the pre-study estimate of self-medication prevalence, i.e., 17.8% or 0.178 [11], and q = 1 − p; z is the standard normal deviation for a 95% confidence interval (1.96) and d is the tolerated sampling error or precision of 5% (d = 0.05). 

Given p = 0.178, q = 1 − p = 0.822, z = 1.96, and d = 0.05, our calculated minimum sample size was 245.

In anticipation of probable loss of some participants due to incomplete data and to increase the power of our results, we predicted and adjusted for a loss of approximately 20% of our participants: adjustment for 20% loss = 100/(100 − 20) = 1.25 [21]. Therefore, our required sample size was 1.25 × 245 = 306.25. We ended up recruiting 308 participants.

### 2.4. Study Procedure

#### Administrative and Ethical Considerations

Administrative clearance was obtained from the Director of MBHD. Ethical clearance was obtained from the Cameroon Baptist Convention Institutional Review Board (Ethical Approval Ref: IRB2016-13). Participants were informed adequately about the study, and its merits and demerits, and interested persons were recruited. Participants had the right to withdraw from the study at any time without any sanctions either directly or indirectly. All records were kept confidential, accessible only to key research personnel. The questionnaires were coded to ensure anonymity and maintain patient confidentiality.

### 2.5. Data Collection

The data collection procedure was explained to all eligible and consenting participants who were diagnosed with RTIs during a routine daily consultation at the outpatient department by the general practitioners of MBHD. A predesigned and validated structured questionnaire was administered by the general practitioners to each participant for approximately 7 min. Questionnaire validity was assessed using face validation by two medical doctors and pilot testing with 5 patients. Questions sought information on socio-demographic characteristics, self-medication use, and sources of self-medication as well as signs or symptoms of respiratory tract infections.

### 2.6. Data Management and Analysis

Questionnaire data was entered into Microsoft Excel 2016, cleaned, and exported to R Studio Version 1.1.383-^©^ 2009–2017 (RStudio, Inc, Boston, MA, USA) for analysis. Antibiotic self-medication in our case was defined as the use of antibiotics to treat symptoms of respiratory tract infection (cough, sneezing, runny nose, sore throat, nasal congestion, nasal breathing, and nasal discharge [22]) without the advice of a physician [8].

The frequency of antibiotic self-medication was obtained. We assessed associations between patients’ socio-demographic and clinical characteristics and antibiotic self-medication using Pearson’s Chi-squared tests for association. The level of significance was set at a *p* value of <0.05.

## 3. Results

### 3.1. Characteristics of the Study Population

A total of 308 participants were recruited in the study. The median age of the participants was 35 years (interquartile range (IQR) 27 to 49). Most of our participants’ (83 (62.9%)) main source of self-medication was the pharmacy (Table 1). The most predominant symptom of our participants was cough only (66.2%, *n* = 204).

### 3.2. Prevalence of Antibiotic Self-Medication

**Figure 1:**
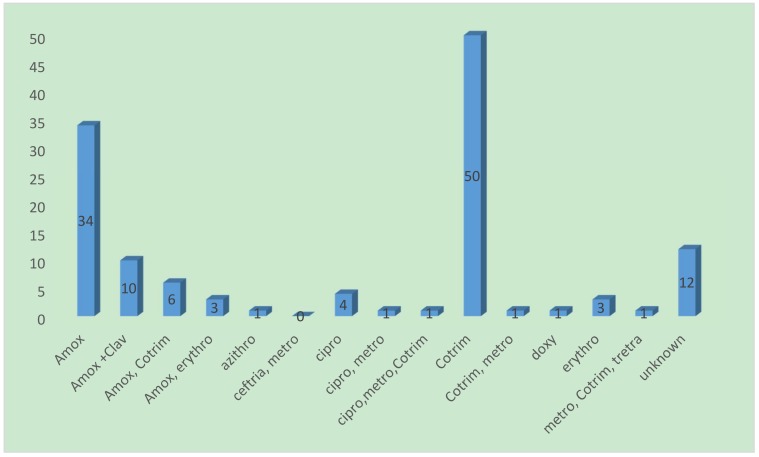
Frequency of various self-medication antibiotics used by participants. Amox = Amoxicillin; Ceftriaxone = Ceftriaxone; Clav = Clavulanic acid; Azithro = Azithromycin; Cotrim = Cotrimoxazole; Metro = Metronidazole; Erythro = Erythromycin; Cipro = Ciprofloxacin; Doxy = Doxycyclin.

A total of 129 (41.9%, 95% CI 36.5% to 47.5%) participants with RTI reported taking antibiotics prior to consultation at the hospital. A majority of the participants (62.0%) reported having obtained the antibiotics from pharmacies (Table 2). Cotrimoxazole and Amoxicillin were the most commonly used antibiotics, having been taken by 38.8% (*n* = 50) and 26.4% (*n* = 34) of the participants, respectively (Figure 1).

### 3.3. Factors Associated with Antibiotic Self-Medication

We found that a history of pulmonary tuberculosis (TB) was significantly associated with antibiotic self-medication (*p*-value = 0.043). There was no association between age, sex, residence, marital status, employment, level of education, alcohol consumption, smoking, and antibiotic self-medication (Table 3).

## 4. Discussion

Antimicrobial resistance (AMR) is an increasingly serious threat to global health and there is a need for prompt action to curb this problem. Misuse of antimicrobials through self-medication in patients with infectious diseases has been reported as a major contributor to AMR. In Cameroon, there is scarcity of data on antibiotic self-medication in common infections such as RTIs which could be self-limiting rather than necessitating antibiotic prescription. To the best of our knowledge this is the first study on antibiotic self-medication in patients with respiratory tract infections in Cameroon. Our aim was to investigate the prevalence and factors associated with antibiotic self-medication in patients with respiratory infections in a faith-based hospital receiving a mass number of patients in the economic capital of Cameroon (Douala).

Our study showed a prevalence of self-medication among patients presenting with respiratory tract infections of 41.9%. In a similar study, Sawair et al. [23] and Muras et al. [10] in Jordan and Poland, respectively, found prevalences of 40.7% and 41.4%. Our finding was lower than those of study reports in Poland (72%) [24], Yemen (78%) [25], Saudi Arabia (78.7%) [26], and Namibia (80%) [27]. A lower prevalence was reported in Indonesia (7.3%) [28] and by the study carried out by Vialle Valentin in five African countries (31.7%) [29]. These disparities in prevalence may be attributed to differences in geographical area, study participants, ethnicity, and health systems, which contribute tremendously to knowledge, behavior, and antibiotic stewardship. In Cameroon, antibiotics are often sold by pharmacists without a doctor’s prescription and from chemists (roadside drug vendors).

We also found that most of the self-prescribed antibiotics were obtained from pharmacists (62.9%) and chemists (18.9%). This is similar to the study by Enas et al. in Saudi Arabia which reported that 53.6% of self-prescribed antibiotics were obtained from pharmacies [30]. These similarities could be attributed to a lack of or inadequately implemented policies on antibiotic use or misuse which are common in low–middle-income settings.

Among the antibiotics received by the patients, the most commonly used antibiotics for self-medication were Cotrimoxazole and Amoxicillin. Our results are in agreement with reported results of the study carried out in Uganda [31]. Just like in most other low- and middle-income countries, these antibiotics are very affordable and are first-line therapies for most respiratory tract infections especially without diagnostic confirmation, thus explaining their predominant use.

We also found that patients with a history of pulmonary tuberculosis were significantly less likely to self-medicate with antibiotics. This may be due to the fact that individuals with a past history of tuberculosis are aware of the severe nature of the disease and are more likely to consult a physician promptly upon onset or relapse of symptoms of RTI. 

Other possible risk factors for antibiotic self-medication such as income and severity or duration of disease were not assessed because they are highly prone to recall bias (our data collected was mainly based on participants self-reporting) and because of the difficulty in measuring factors such as disease severity when it involves participants with multiple respiratory conditions. In addition, salary scales are not standardized or readily available in most low–middle-income countries which makes it difficult to calculate the income level of participants. The hypothesis generated herein should be tested in future studies which adjust for the above potentially confounding factors.

### 4.1. Study Limitations

Some of the limitations to have in mind when interpreting these results may include the following: This study was a cross-sectional study carried out in an urban setting and in a single institution, thereby limiting the generalizability of our results to other settings. Also, our study was conducted in a faith-based facility; hence, our findings might be different from those in private and public healthcare facilities.

Also, a significant number of patients who engage in antibiotic self-medication do not end up consulting the hospital and, as such, this study might underestimate the true prevalence of self-medication in the community.

### 4.2. Policy Implications

Policies on antibiotic stewardship should be enforced or re-enforced, detailing the signs and symptoms highly suggestive of bacterial respiratory tract infection before antibiotic use as opposed to very common viral infections. This could be ensured by physicians and pharmacists to minimize unnecessary antibiotic use. In addition, educational campaigns for the general population on antibiotic use and the downsides of antibiotic self-medication at the individual, community, national, and global level should be instituted.

## 5. Conclusions

This study revealed that self-medication with antibiotics for respiratory tract infections is a common practice in Cameroon, which should be addressed. Awareness of the disadvantages of antibiotic self-medication without a diagnosis, like with the case of patients with a history of pulmonary TB, reduces antibiotic self-medication. We therefore recommend the education of pharmacy personnel and the general population on the dangers associated with antibiotic self-medication for RTI. Also, further research should be carried out to establish highly sensitive and specific point-of-care tests which can be used for rapid diagnosis before antibiotic use.

## Figures and Tables

**Table 1:** Characteristics of the study population.

Variable	Frequency (%)
Age (years)	
Median (IQR) **	35 (27–49)
Sex	
Male	138 (44.8%)
Female	170 (55.2%)
Occupation	
Employed	152 (49.4%)
Unemployed	156 (50.6%)
Marital Status	
Married	140 (45.6%)
Single	133 (43.3%)
Widowed	25 (8.1%)
Divorced	9 (2.9%)
Level of Education	
None	29 (9.4%)
Primary	102 (33.1%)
Secondary/High school	124 (40.3%)
Tertiary/University	53 (17.2%)
Residence	
Urban	247 (80.2%)
Rural	61 (19.8%)
Alcohol(yes)	150 (48.7%)
Smoking(yes)	32 (10.4%)
History of pulmonary tuberculosis (yes)	26 (8.4%)

** IQR: Interquartile range.

**Table 2:** The main sources of self-medication.

Variables	Frequency(%)
Source of self-medication antibiotic	
Home	10 (7.7%)
Friends/relatives	14 (10.9%)
Pharmacy	80 (62.0%)
Chemist	25 (19.4%)

**Table 3:** Factors determining antibiotic self-medication.

Variable	Antibiotic Self-Medication Frequency (%)			
	Yes	No	Total	*p*-value
**Age (years)**				0.289
≤35	62 (39%)	97 (61%)	159 (51.6%)	
>35	67 (45%)	82 (55%)	149 (48.4%)	
**Gender**				0.266
Male	53 (38.4%)	85 (61.6%)	138 (44.8%)	
Female	76 (44.7%)	94 (55.3%)	170 (55.2%)	
**Residence**				0.318
Urban	100 (40.5%)	147 (59.5%)	247 (80.2%)	
Rural	29 (47.5%)	32 (52.5%)	61 (19.8%)	
**Level of Education**				0.464
Primary and above	115(41.2%)	164(58.8%)	279(90.6%)	
None	14(48.3%)	15(51.7%)	29(9.4%)	
**Occupation**				0.539
Employed	61 (40.1%)	91 (59.9%)	152 (49.4%)	
Unemployed	68 (43.6%)	88 (56.4%)	156 (50.6%)	
**Marital status**				0.462
Married/cohabiting	62 (44.3%)	78 (55.7%)	140 (45.6%)	
Single/divorced/widowed	67 (40.1%)	100 (59.9%)	167 (54.4%)	
**Alcohol**				0.515
Yes	60 (40.0%)	90 (60.0%)	150 (48.7%)	
No	69 (43.7%)	89 (56.3%)	158 (51.3%)	
**Smoking**				0.879
Yes	13 (40.6%)	19 (59.4%)	26 (8.4%)	
No	116 (42.0%)	160 (58.0%)	276 (89.6%)	
History of PTB *				0.043
Yes	6 (23.1%)	20 (76.9%)	26 (8.4%)	
No	123 (43.6%)	159 (56.4%)	282 (91.6%)	

* PTB = Pulmonary Tuberculosis; NB: Pearson’s Chi square test of independence was the test statistic used for the above associations.
